# Telehealth Service Delivery in Medicaid Home- and Community-Based Services for People with Intellectual and Developmental Disabilities

**DOI:** 10.5195/ijt.2022.6478

**Published:** 2022-06-03

**Authors:** Carli Friedman

**Affiliations:** 1 CQL | The Council on Quality and Leadership, Towson, MD, USA

**Keywords:** Long-Term Services and Supports (LTSS), Medicaid Home- and Community-Based Services (HCBS), People with intellectual and developmental disabilities, Telehealth

## Abstract

Medicaid Home- and Community-Based Services (HCBS) 1915(c) waivers are the largest funding mechanism for Long-Term Supports and Services (LTSS) for people with intellectual and developmental disabilities (IDD) in the United States. This study's aim was to examine telehealth service provision in Medicaid HCBS waivers for people with IDD. We analyzed fiscal year 2021 Medicaid HCBS waivers for people with IDD and emergency Appendix K authorizations (2020-2022) to examine permanent and temporary use of telehealth, respectively. The overwhelming majority of waivers (98.1%) temporarily permitted the use of telehealth service delivery for people with IDD. However, only a fraction (27.6%) permanently included the use of telehealth for people with IDD. The most prevalent types of services that permitted telehealth service delivery were: employment, day, and prevocational services; clinical and therapeutic services; and in-home and residential supports. When developing and implementing telehealth, it is important to consider the needs of people with IDD.

Long-Term Services and Supports (LTSS) are facility- or community-based services designed to serve people in the United States who need assistance to care for themselves as a result of functional limitations, age, and/or disability, such as people with intellectual and developmental disabilities (IDD). While originally, states could only use Medicaid to fund LTSS in institutional settings, in 1981 Home- and Community-Based Services (HCBS) 1915(c) waivers were introduced, allowing states to wave the three main provisions of the Social Security Act: state-wideness, comparability, and income and resource rules. As a result, states are able to create community-based LTSS tailored to specific populations that would otherwise require institutional care. States have flexibility to determine waiver eligibility, what types of services are covered, and how those services are provided. As a result of the preferences of people with IDD, and the improved outcomes and cost effectiveness of community living, Medicaid HCBS waivers have become the largest funding mechanism for the LTSS of people with IDD (Braddock et al., 2017; Friedman, 2020). In FY 2021, $43.2 billion of spending was projected for the HCBS of 861,038 people with IDD (Friedman, 2022).

The COVID-19 pandemic significantly disrupted HCBS service provision for people with IDD (Nygren & Lulinski, 2020). As a result of the increased threat COVID-19 represents to people with IDD, including for those who live in congregate settings, many people with IDD have been forced to stay home and isolate (Abrams et al., 2020; Bradley, 2020; Clift et al., 2021; Embregts et al., 2022; Gleason et al., 2021; Kompaniyets et al., 2021; Landes et al., 2020; Luterman, 2020; Pettinicchio et al., 2021; Williamson et al., 2020). In addition, many HCBS providers were required to limit or close services due to government safety orders (ANCOR Foundation & United Cerebral Palsy, 2021; Avalere Health, 2020; Bradley, 2020; Ervin & Hobson-Garcia, 2020). The pandemic also intensified staffing shortages, making in-person service delivery more challenging (ANCOR Foundation & United Cerebral Palsy, 2021). These factors lead to an increased need for telehealth and remote technologies, which although not new technologies, had not widely been used with people with IDD, especially given technology disparities for this population (Brewer et al., 2010; Lussier-Desrochers et al., 2017; Perry et al., 2009).

During the COVID-19 pandemic, many health care providers shifted to telehealth (Galewitz, 2020). The use of telehealth for service delivery also increased for service provision for people with disabilities (ANCOR Foundation & United Cerebral Palsy, 2021; Scheffers et al., 2021). In fact, approximately 40% of people with disabilities used telehealth in 2021 (Friedman & VanPuymbrouck, 2021). People with cognitive disabilities were also more likely to use telehealth than people with other disabilities in 2021 (Friedman & VanPuymbrouck, 2021). Despite this, less is known about telehealth service delivery specifically in HCBS for people with IDD. For these reasons, the aim of this study was to examine telehealth service provision in Medicaid HCBS waivers for people with IDD. To do so, we analyzed fiscal year (FY) 2021 Medicaid HCBS 1915(c) IDD waivers and Appendix K amendments to HCBS 1915(c) IDD waivers (2020-2022).

## METHODS

### PERMANENT ALLOCATION OF TELEHEALTH IN HCBS 1915(C) WAIVERS^1^

Medicaid HCBS 1915(c) waivers were obtained from the Centers for Medicare and Medicaid Services' (CMS) Medicaid.gov website between December 2021 and January 2022. Inclusion criteria required that waivers be 1915(c), serve people with IDD (developmental disabilities, intellectual disabilities, and/or autism), and include 2021. Most states used the state FY (July 1, 2020 to June 30, 2021), however, others used the federal FY (October 1, 2020 to September 30, 2021), or the 2021 calendar year (January 1, 2021 to December 31, 2021) – we use the term FY for consistency. This process resulted in 107 FY 2021 HCBS 1915(c) waivers for people with IDD from 44 states and the District of Columbia.

In their HCBS waivers, states are required to define each service they will provide to waiver participants, including the service's scope, limitations, and other requirements for service provision (Centers for Medicare and Medicaid Services, 2019). We reviewed each service definition across the 107 waivers to determine which services specified that the service could be provided via real-time synchronous audio/visual telehealth. We extracted those services that permitted telehealth and analyzed their service definitions using thematic analysis (Braun & Clarke, 2006) to determine major and minor themes.

### TEMPORARY PANDEMIC ALLOCATION OF TELEHEALTH (APPENDIX K)^2^

During the pandemic states could make temporary emergency changes to their 1915(c) waiver programs through *Appendix K: Emergency Preparedness and Response Waivers* (Centers for Medicare and Medicaid Services, n.d.). We obtained Appendix Ks from the Medicaid.gov website on April 7, 2022. Inclusion criteria required that Appendix Ks applied to 1915(c) waivers for people with IDD (developmental disabilities, intellectual disabilities, and/or autism), and be due to the COVID-19 pandemic (rather than natural disasters). This process resulted in a collection of 294 Appendix Ks from 44 states and the District of Columbia.

In Appendix K, states are required to document the ways they will temporarily change their HCBS 1915(c) waivers during the pandemic. We reviewed this information to determine which services specified they could temporarily be provided via real-time synchronous audio/visual telehealth. We extracted those services that permitted telehealth in the Appendix Ks and analyzed their service definitions using thematic analysis to determine major and minor themes.

## RESULTS

### PERMANENT ALLOCATION OF TELEHEALTH IN HCBS WAIVERS

In FY 2021, 185 services provided by 29 waivers (27.6%) from 18 states and the District of Columbia (42.2%) permanently included the use of telehealth for HCBS IDD service provision. Of those services that allowed telehealth, the most prevalent types of services were employment, day, and prevocational services (40.0%); in-home and residential supports (23.2%); and clinical and therapeutic services (17.8%; Table 1). While half (49.7%, *n* = 92) of the services did not identify the specific telehealth delivery method, of those that did, 7.0% (*n* = 13) specified telehealth by telephone only, 5.4% (*n* = 10) by video only, and 37.8% (*n* = 70) both telephone and video.

**Table 1:** Telehealth Service Delivery for People with IDD

Category	Permanent (1915(c) waiver 2021; *n* = 185)		Temporary during the pandemic (Appendix K 2020-2022; *n* = 1,392)	
	*n*	%	*n*	%
Employment, day, and prevocational	74	40.0%	431	31.0%
In-home and residential supports	43	23.2%	218	15.7%
Clinical and therapeutic	33	17.8%	416	29.9%
Crisis and respite	12	6.5%	78	5.6%
Community integration supports	7	3.8%	87	6.3%
Support coordination	7	3.8%	34	2.4%
Family support services	4	2.2%	27	1.9%
Peer mentorship	4	2.2%	7	0.5%
Specialized medical equipment and assistive technology	1	0.5%	78	5.6%
Adult day health	0	0.00%	16	1.1%

In terms of themes across the services, three-quarters of the time (78.4%, *n* = 145) services required that the use of telehealth be selected by the person with IDD, specifically through informed choice. For example, Maryland Community Pathways waiver's (MD0023's) “personal supports” service said,

*The use of virtual supports to provide direct support has been agreed to by the [waiver] participant and their team and is outlined in the Person-Centered Plan; Participants must have an informed choice between in person and virtual supports; Virtual supports cannot be the only service delivery provision for a participant seeking the given service; and Participants must affirmatively choose virtual service provision over in-person supports.*

In addition, 34.1% of services (*n* = 63) clarified that telehealth cannot be used for provider convenience.

It was also common (62.2%, *n* = 115) for services to specify that the platform or system used for telehealth must be HIPAA compliant. For example, Colorado Children's Extensive Support Waiver's (CO4180's) “movement therapy – bachelors” service noted,

*Each provider of the telehealth service delivery option must demonstrate policies and procedures that include they have a HIPAA compliant platform. HIPAA compliance will be reviewed regularly through the Colorado Department of Public Health and Environment (CDPHE) survey and monitoring process. Each provider will sign an attestation that they are using a HIPAA compliant platform for the Telehealth service component. The provider requirements and assurances regarding HIPAA have been approved by the states HIPAA Compliance Officer.*

Moreover, more than half of the services (61.1%, *n* = 113) explicitly required that the use of telehealth honor people with IDD's rights to privacy. For example, Minnesota's Developmental Disabilities Waiver's (MN0061's) “family training and counseling” service said, there must be “*respect*” and telehealth delivery must “*maintain the person's privacy at all times, including when the person is in settings typically used by the general public*.” In addition, a number of services (9.2%, *n* = 17) went further by specifying telehealth is prohibited in bathrooms.

Services also emphasized that telehealth should be used for the purpose of promoting people with IDD's outcomes (49.2%, *n* = 91), including their community integration, engagement, and achievement of goals. For example, Connecticut Comprehensive Supports Waiver's (CT0437's) “individualized home supports” service specified it “*Can include face-to-face interactions including Face Time or comparable technology (such as IPAD, IPHONE) that are designed to promote ongoing engagement of waiver participants towards the participant's personal goals.*” In addition, many services (22.2%, *n* = 41) said the use of telehealth for service provision cannot result in people with IDD becoming isolated from their communities or other people. For example, New York OPWDD Comprehensive Renewal Waiver's (NY0238's) “day habilitation - group - FFS” service required,

*The remote supports do not isolate the [waiver] participant from the community or interacting with people without disabilities. The participant has other opportunities for integration in the community via the other Waiver program services the participant receives and are provided in community settings.*

About one-quarter (24.3%, *n* = 45) also emphasized systems must be in place to ensure the health and safety of people with IDD while telehealth is being utilized. For example, Oklahoma In-Home Supports Waiver for Adults' (OK0343's) “occupational therapy” service noted, “*Telehealth providers will ensure member [waiver participant] health and safety by contacting a member's caregiver in the event a health or safety issue becomes evident during a telehealth session*.”

About half of services (51.9%, *n* = 96) put limitations on the ratio between telehealth and in-person service provision. For example, District of Columbia's People with IDD Waiver's (DC0307's) “in-home supports” service said, *“In Home Supports services by phone or other technological means cannot exceed 20% of the total In Home Supports services that the person receives each week*.” While most services with this requirement simply said people must have access to both in-person and telehealth, there were others (14.1%, *n* = 26) that said no more than 20% of weekly service provision could be via telehealth, three services (1.6%) said telehealth could only be used in times of crisis, and two services (1.1%) said telehealth could only be used for two hours maximum a day.

While about one-quarter of services (23.8%, *n* = 44) specified that people with IDD should be supported to make telehealth more accessible for them, 35.7% of waivers (*n* = 66) noted that telehealth can only be used for service delivery if people with IDD are able to use telehealth with minimal help. For example, NY0238's “respite - self-directed - FFS” service said, “*To be appropriate for the person, the remote delivery of services must be able to be effectuated via verbal prompting only.*” In addition, 23.8% (*n* = 44) of services required assessments be conducted, prior to implementation, to determine if the person with IDD is able to use telehealth. For example, Colorado Supported Living Services waiver's (CO0293's) “mentorship” service specified,

*Members [waiver participants] who require hands on assistance during the provision of the service must receive services in-person. In order to ensure the health and safety of members, case managers and providers must assess the appropriateness of virtual services with member. If it is determined that hands-on assistance is required, virtual services may not be provided. This process will be outlined in each provider's policies and procedures.*

In terms of other requirements, 23.8% of services (*n* = 44) prohibited out-of-state providers from being reimbursed for telehealth, 23.8% of services (*n* = 44) noted contingency plans must be in place in case technology fails, 19.5% of services (*n* = 36) explicitly said the costs of telehealth equipment were excluded, and 10.3% of services (*n* = 19) required providers to have policies outlining the use of telehealth. For example, MD0023's “employment services - follow along supports” specified:

*The provider must develop and maintain written policies, train direct support staff on those policies, and advise [waiver] participants and their person- centered planning team regarding those policies that address: Identifying whether the participant's needs, including health and safety, can be addressed safely via virtual supports; Identifying individuals to intervene (such as uncompensated caregivers present in the participant's home), and ensuring they are present during provision of virtual supports in case the participant experiences an emergency during provision of virtual supports; Processes for requesting such intervention if the participant experiences an emergency during provision of virtual supports, including contacting 911 if necessary… How the provider will ensure the participant's rights of privacy, dignity and respect, and freedom from coercion and restraint; How the provider will ensure the virtual supports used meets applicable information security standards; and, How the provider will ensure its provision of virtual supports complies with applicable laws governing individuals' right to privacy.*

### TEMPORARY PANDEMIC ALLOCATION OF TELEHEALTH (APPENDIX K)

During the COVID-19 pandemic (March 2020 through April 2022), 41 states and the District of Columbia (93.3% of states) temporarily permitted telehealth service delivery through Appendix K in 105 different HCBS 1915(c) waivers for people with IDD (98.1% of waivers). Of the 105 waivers which permitted telehealth service delivery, 21 waivers did not provide information in their Appendix Ks about *which* specific services could be provided by telehealth other than to say personal care services that only require verbal cueing, in-home habilitation, and/or “other” could be provided by telehealth. However, among the 83 waivers that provided details about which services could be delivered by telehealth in Appendix K, a total of 1,392 different services could be provided by telehealth. The three most prevalent types of services which temporarily permitted telehealth were: employment, day, and prevocational services (31.0%); clinical and therapeutic services (29.9%); and in-home and residential supports (15.7%; Table 1).

Among these 1,392 services, 168 services did not provide further details other than the service name; the remaining 1,224 services were analyzed for themes. In terms of telehealth delivery method, 53.7% of services (*n* = 657) specified the service could be provided by both telephone and video, 0.3% (*n* = 4) by telephone only, and 46.0% (*n* = 563) did not specify method.

Appendix K telehealth service delivery descriptions commonly specified that the platform or system used for telehealth must be HIPAA compliant (32.7%, *n* = 400). For example, Minnesota's Developmental Disabilities waiver's (MN0061's) ‘Homemaker’ service said:

*Telephonic or other remote methods of service delivery will be conducted in accordance with HIPAA requirements, to the extent possible, but with recognition that the Office of Civil Rights is not enforcing certain requirements for good faith communications during the period of the national emergency.*

Many services also noted that telehealth should promote the health and safety of people with IDD (31.7%, *n* = 388). For example, Idaho's Developmental Disabilities waiver's (ID0076's) “Support Broker Services” said it “*may be delivered via electronic methods if the service can be safely and effectively delivered via electronic methods.*”

Approximately one-tenth of services (10.8%, *n* = 132) required telehealth either be approved by the person with IDD's support coordinator and/or documented in their individual support plan in order to be permitted. For example, Connecticut's Home and Community Supports Waiver for Persons with Autism's (CT0993's) “Life Skills Coach Direct Hire” service said, “*Life Coach services may be provided electronically or telephonically, with approval from the care manager.*”

A number of services (8.4%, *n* = 103) required that the use of telehealth by selected by the person with IDD through informed choice. For example, California HCBS Waiver for Californians with Developmental Disabilities' (CA0336's) “Physical Therapy” service documented,

*Prior to the delivery of a service by electronic communications, the service provider must notify the regional center that the consumer(s) or representative agrees to remote or virtual services in lieu of in-person services. The regional center shall send a follow-up letter to the family, in the family's preferred language, confirming that at the family's request, virtual or remote services will be provided in lieu of in-person services.*

Some services noted telehealth service delivery could only be used if the person with IDD needs minimal support (7.3%, *n* = 89). Meanwhile, other services noted telehealth should only be used as a last resort (6.9%, *n* = 85). For example, Indiana Community Integration and Habilitation Waiver's (IN0378's) “Respite” service, “*…may use telemedicine as a last resort option, only with individuals who need only verbal prompting and guidance, and must relate to an individualized need or interest.*” Additional themes included that an assessment must be conducted prior to people with IDD being able to use telehealth (1.5%, *n* = 18), privacy rights be respected (1.1%, *n* = 13), and prohibiting the use of telehealth in bathrooms (0.8%, *n* = 10).

## DISCUSSION

The use of telehealth rapidly increased during the COVID-19 pandemic (Galewitz, 2020). The aim of this study was to examine telehealth service provision in Medicaid HCBS waivers for people with IDD. In doing so, we found the overwhelming majority of HCBS 1915(c) waivers (98.1%) temporarily permitted the use of telehealth service delivery for people with IDD via Appendix K. However, only a fraction of HCBS 1915(c) waivers (27.6%) permanently included the use of telehealth for people with IDD. Almost eight times the number of services could temporarily be delivered by telehealth (*n* = 1,392) than permanently (*n* = 185). While these findings suggest a significant increase in the use of telehealth in HCBS IDD waivers through the implementation of Appendix K, as Appendix K is a temporary authority, they also suggest the use of telehealth service delivery for IDD HCBS will significantly diminish when the public health emergency ends. As such, we believe states should amend their HCBS 1915(c) waivers to permanently expand the number of services which permit telehealth delivery. This is especially pertinent as telehealth can expand people's access to care and may help reduce health disparities (Dinesen et al., 2016).

While telehealth service delivery was permitted by a range of services, such as community integration supports, crisis and respite, and in-home and residential supports, the most prominent service category to permit telehealth, both temporarily and permanently, was employment, day, and prevocational services. While we expected a higher utilization rate for clinical and therapeutic services, the use of telehealth for employment, day, and prevocational services is not surprising as many of these in-person settings were closed during the pandemic because of safety requirements and regulations (ANCOR Foundation & United Cerebral Palsy, 2021; Bradley, 2020; Luterman, 2020). As a result of this impact, states likely introduced telehealth service delivery to supplement or make up for in-person restrictions, especially since employment, day, and prevocational services can offer people with IDD a sense of belonging, achievement, and structure (Scheffers et al., 2021).

We believe the focus on informed choice, outcomes, and privacy are important practices for telehealth for people with IDD that should be expanded not only in HCBS, but also anywhere telehealth is used with people with IDD. The requirements that the use of telehealth does not result in people with IDD becoming isolated is also important given that COVID-19 resulted in increased isolation of people with IDD (ANCOR Foundation & United Cerebral Palsy, 2021), and because people with IDD, especially those who receive HCBS, have the right to community integration according to the HCBS Final Settings Rule (Centers for Medicare and Medicaid Services, 2014) and the *Olmstead v. L.C.* (1999) Supreme Court decision, which is based on the Americans with Disabilities Act's (ADA's; 1990) integration mandate.

A number of services prohibited people with IDD from receiving telehealth service delivery if they were not able to use telehealth independently. Doing so will significantly limit the number of people with IDD who are able to use telehealth, which could have been utilized to expand and supplement people's access to services. There is no reason people with IDD should be prohibited from accommodations, such as the assistance of a direct support professional, especially given the rights they are entitled to according to the ADA. Moreover, there was relatively little mention of telehealth accessibility among the service definitions, which is problematic because telehealth is inaccessible for many people with IDD as many services and platforms have failed to consider the needs of this population (Dobransky & Hargittai, 2016; Krysta et al., 2021; Young & Edwards, 2020). In fact, allowing staff or others to assist people with IDD while using telehealth is one mechanism to make up for the lack of accessibility of this form of service delivery (Valdez et al., 2021). As such, we recommend states not only draw more attention to the accessibility of telehealth in their HCBS waivers, but also remove the requirements of independence for telehealth utilization. When doing so, it is important to note that people with disabilities are significantly less likely to have access to technology, the internet, and smartphones than nondisabled people (Anderson & Perrin, 2017); in fact, we believe this is likely why there was a lack of permanent uptake of telehealth service delivery. As such, technology disparities may serve as a barrier to telehealth for people with IDD and must be remedied.

## LIMITATIONS

When interpreting the findings from this study, a number of limitations should be noted. While our analysis of HCBS waiver and Appendix K policies explored which states, waivers, and services permitted the use of telehealth, the data did not include information on how many people with IDD were able to actually make use of telehealth or their experiences with telehealth. Moreover, we analyzed major and minor themes across the service definitions for those services which specified permitting telehealth in the waivers and through Appendix K; however, states may have used other mechanisms outside of HCBS and appendix K to offer telehealth and/or implement requirements for telehealth service provision. For example, many of the service definitions did not mention HIPAA compliance requirements; yet, HIPAA requirements would certainly apply, especially for clinical and therapeutic services. We believe these limitations also represent opportunities for future study.

## CONCLUSION

People with IDD have historically faced technology disparities (Anderson & Perrin, 2017; Lussier-Desrochers et al., 2017). As such, telehealth and remote technologies have been less common for this population. This study found, permanently, 18 states and the District of Columbia (42.2%) permitted 185 services to use telehealth in 2021, and 41 states and the District of Columbia (93.3%) temporarily permitted 1,392 services to use telehealth during the pandemic (2020-2022). While telehealth has the potential to reduce health care disparities, barriers to access and utilization, such as a lack of accessibility or requirements of independence, could serve to further increase disparities (Centers for Disease Control and Prevention, 2020). As such, when developing and implementing telehealth, including in HCBS, it is important to consider the needs of people with IDD.

## Footnotes

1 Quoted content from the respective 1915(c) waivers can be located in the state waiver list at https://www.medicaid.gov/medicaid/section-1115-demo/demonstration-and-waiver-list/index.html

2 Quoted content from the respective state Appendix K demonstrations can be located in HCBS 1915(c) Appendix K Data table at https://www.medicaid.gov/resources-for-states/disaster-response-toolkit/home-community-based-services-public-health-emergencies/emergency-preparedness-and-response-for-home-and-community-based-hcbs-1915c-waivers/index.html

## References

[B1] Abrams, H.R., Loomer, L., Gandhi, A., & Grabowski, D. C. (2020). Characteristics of US nursing homes with COVID-19 cases. *Journal of the American Geriatrics Society*, 68(8), 1653–1656. 10.1111/jgs.16661 32484912 PMC7300642

[B2] Americans With Disabilities Act of 1990, Pub. L. No. 101-336, 104 Stat. 328, (1990).

[B3] ANCOR Foundation, & United Cerebral Palsy. (2021). The case for inclusion 2021: A special report on the sustainability of community disability services in America. https://caseforinclusion.org/application/files/2416/1376/5849/Case_for_Inclusion_2021_Special_Report.pdf

[B4] Anderson, M., & Perrin, A. (2017). Disabled Americans are less likely to use technology. https://www.pewresearch.org/fact-tank/2017/04/07/disabled-americans-are-less-likely-to-use-technology/

[B5] Avalere Health. (2020). Impact of COVID-19 on organizations serving individuals with intellectual and developmental disabilities. American Network of Community Options and Resources (ANCOR). https://www.ancor.org/sites/default/files/impact_of_covid-19_on_organizations_serving_individuals_with_idd.pdf

[B6] Braddock, D., Hemp, R., Tanis, E.S., Wu, J., & Haffer, L. (2017). The state of the states in intellectual and developmental disabilities: 2017 (11th ed.). The American Association on Intellectual and Developmental Disabilities.

[B7] Bradley, V. J. (2020). How COVID-19 may change the world of services to people with intellectual and developmental disabilities. *Intellectual and Developmental Disabilities*, 58(5), 355–360. 10.1352/1934-9556-58.5.355 33032314

[B8] Braun, V., & Clarke, V. (2006). Using thematic analysis in psychology. *Qualitative Research in Psychology*, 3(2), 77–101. 10.1191/1478088706qp063oa

[B9] Brewer, J.L., Taber-Doughty, T., & Kubik, S. (2010). Safety assessment of a home-based telecare system for adults with developmental disabilities in Indiana: A multi-stakeholder perspective. *Journal of telemedicine and telecare*, 16(5), 265–269. 10.1258/jtt.2010.090902 20501628

[B10] Centers for Disease Control and Prevention. (2020). Using telehealth to expand access to essential health services during the COVID-19 pandemic. https://www.cdc.gov/coronavirus/2019-ncov/hcp/telehealth.html#print

[B11] Centers for Medicare and Medicaid Services. (2014). Medicaid Program; State Plan Home and Community-Based Services, 5-year period for waivers, provider payment reassignment, and Home and Community-Based Setting requirements for Community First Choice and Home and Community-Based Services (HCBS) waivers (CMS 2249-F/2296-F). Author. 24443765

[B12] Centers for Medicare and Medicaid Services. (2019). Application for a §1915(c) Home and Community-Based Waiver [Version 3.6]: Instructions, technical guide, and review criteria. https://www.hhs.gov/guidance/sites/default/files/hhs-guidance-documents/instructions_technicalguide_v3.6.pdf

[B13] Centers for Medicare and medicaid Services. (n.d.). 1915(c) Home and Community-Based Services waiver instructions and technical guidance: Appendix K: Emergency preparedness and response. https://www.medicaid.gov/state-resource-center/downloads/1915c-appendix-k-instructions.pdf

[B14] Clift, A.K., Coupland, C.A., Keogh, R.H., Hemingway, H., & Hippisley-Cox, J. (2021). COVID-19 mortality risk in Down syndrome: Results from a cohort study of 8 million adults. *Annals of Internal Medicine*, 174(4), 572–576. 10.7326/M20-4986 33085509 PMC7592804

[B15] Dinesen, B., Nonnecke, B., Lindeman, D., Toft, E., Kidholm, K., Jethwani, K., Young, H.M., Spindler, H., Oestergaard, C.U., & Southard, J. A. (2016). Personalized telehealth in the future: A global research agenda. *Journal of Medical Internet Research*, 18(3), e5257. 10.2196/jmir.5257 PMC4795318 26932229

[B16] Dobransky, K., & Hargittai, E. (2016). Unrealized potential: Exploring the digital disability divide. *Poetics*, 58, 18–28. 10.1016/j.poetic.2016.08.003

[B17] Embregts, P.J., van den Bogaard, K.J., Frielink, N., Voermans, M.A., Thalen, M., & Jahoda, A. (2022). A thematic analysis into the experiences of people with a mild intellectual disability during the COVID-19 lockdown period. *International Journal of Developmental Disabilities*, 15(2), 168–196. 10.1080/20473869.2020.1827214 PMC9351556 35937180

[B18] Ervin, D.A., & Hobson-Garcia, D. (2020). Community supports and COVID-19: Self-determination in a pandemic. *Intellectual and Developmental Disabilities*, 58(6), 453–457. 10.1352/1934-9556-58.6.453 33290529

[B19] Friedman, C. (2020). There's no place like home: A national study of how people with intellectual and/or developmental disabilities and their families choose where to live. The Arc of the United States, & CQL | The Council on Quality and Leadership. https://futureplanning.thearc.org/assets/CFP_Housing_Survey_Technical_Report-80e6eb718c816d07a15a9972df06a6e73b1393d5b56ae145acc058fce243cd93.pdf

[B20] Friedman, C. (2022). Medicaid Home- and Community-Based Services for people with intellectual and developmental disabilities. Manuscript submitted for publication.

[B21] Friedman, C., & VanPuymbrouck, L. (2021). Telehealth use by persons with disabilities during the COVID-19 pandemic. *International Journal of Telerehabilitation*, 13(2). 10.5195/ijt.2021.6402 PMC9098125 35646237

[B22] Galewitz, P. (2020). Telemedicine surges, fueled by coronavirus fears and shift in payment rules. Kaiser Health News. https://khn.org/news/telemedicine-surges-fueled-by-coronavirus-fears-and-shift-in-payment-rules/

[B23] Gleason, J., Ross, W., Fossi, A., Blonsky, H., Tobias, J., & Stephens, M. (2021). The devastating impact of COVID-19 on individuals with intellectual disabilities in the United States. *NEJM Catalyst Innovations in Care Delivery*, 2(2). 10.1056/CAT.21.0051

[B24] Kompaniyets, L., Pennington, A.F., Goodman, A.B., Rosenblum, H.G., Belay, B., Ko, J.Y., Chevinsky, J.R., Schieber, L.Z., Summers, A.D., & Lavery, A. M. (2021). Underlying medical conditions and severe illness among 540,667 adults hospitalized with COVID-19, March 2020-March 2021. *Preventing Chronic Disease*, 18. 10.5888/pcd18.210123 PMC8269743 34197283

[B25] Krysta, K., Romańczyk, M., Diefenbacher, A., & Krzystanek, M. (2021). Telemedicine treatment and care for patients with intellectual disability. *International Journal of Environmental Research and Public Health*, 18(4), 1746. 10.3390/ijerph18041746 33670152 PMC7916831

[B26] Landes, S.D., Turk, M.A., Formica, M.K., McDonald, K.E., & Stevens, J. D. (2020). COVID-19 outcomes among people with intellectual and developmental disability living in residential group homes in New York State. *Disability and Health Journal*, 13(4), 100969. 10.1016/j.dhjo.2020.100969 32600948 PMC7311922

[B27] Lussier-Desrochers, D., Normand, C.L., Romero-Torres, A., Lachapelle, Y., Godin-Tremblay, V., Dupont, M.-È., Roux, J., Pépin-Beauchesne, L., & Bilodeau, P. (2017). Bridging the digital divide for people with intellectual disability. *Cyberpsychology: Journal of Psychosocial Research on Cyberspace*, 11(1). 10.5817/CP2017-1-1

[B28] Luterman, S. (2020). The neglect of disability care. The American Prospect. https://prospect.org/familycare/the-neglect-of-disability-care/

[B29] Nygren, M.A., & Lulinski, A. (2020). State of the science on COVID-19 and people with IDD. American Association on Intellectual and Developmental Disabilities. https://www.aaidd.org/docs/default-source/publication/state-of-the-science-on-covid-19-and-people-with-idd---dec-2020.pdf?sfvrsn=25893421_0

[B30] Olmstead v. LC, 527 US 581 (Supreme Court 1999).

[B31] Perry, J., Beyer, S., & Holm, S. (2009). Assistive technology, telecare and people with intellectual disabilities: ethical considerations. *Journal of Medical Ethics*, 35(2), 81–86. 10.1136/jme.2008.024588 19181877

[B32] Pettinicchio, D., Maroto, M., Chai, L., & Lukk, M. (2021). Findings from an online survey on the mental health effects of COVID-19 on Canadians with disabilities and chronic health conditions. *Disability and Health Journal*, 14(3), 101085. 10.1016/j.dhjo.2021.101085 33744158 PMC9760304

[B33] Scheffers, F., van Vugt, E., & Moonen, X. (2021). Assessing the quality of support and discovering sources of resilience during COVID-19 measures in people with intellectual disabilities by professional carers. *Research in Developmental Disabilities*, 111, 103889. 10.1016/j.ridd.2021.103889 33578230 PMC9758889

[B34] Valdez, R.S., Rogers, C.C., Claypool, H., Trieshmann, L., Frye, O., Wellbeloved-Stone, C., & Kushalnagar, P. (2021). Ensuring full participation of people with disabilities in an era of telehealth. *Journal of the American Medical Informatics Association*, 28(2), 389–392. 10.1093/jamia/ocaa297 33325524 PMC7717308

[B35] Williamson, E.J., Walker, A.J., Bhaskaran, K., Bacon, S., Bates, C., Morton, C.E., Curtis, H.J., Mehrkar, A., Evans, D., & Inglesby, P. (2020). Factors associated with COVID-19-related death using OpenSAFELY. *Nature*, 584(7821), 430–436. 10.1038/s41586-020-2521-4 32640463 PMC7611074

[B36] Young, D., & Edwards, E. (2020). Telehealth and disability: Challenges and opportunities for care. National Health Law Program. https://healthlaw.org/telehealth-and-disability-challenges-and-opportunities-for-care/

